# Malignant transformation of desmoplastic infantile tumors in a non-infant: a case report

**DOI:** 10.3389/fonc.2023.1272831

**Published:** 2023-11-27

**Authors:** Yan Yang, Bin Tian, Xuzhu Chen, Xin Liu, Shiguang Li

**Affiliations:** ^1^ Department of Radiology, The Second People’s Hospital of Guiyang, Guiyang, China; ^2^ Department of Radiology, Beijing Tiantan Hospital, Capital Medical University, Beijing, China; ^3^ Department of Pathology, Beijing Tiantan Hospital, Capital Medical University, Beijing, China

**Keywords:** desmoplastic infantile tumors, malignant transformation, computed tomography, magnetic resonance imaging, case report

## Abstract

Desmoplastic infantile tumors (DITs) are rare benign intracranial tumors in infants with benign biological behavior and rare malignant transformation characteristics. We present a DIT case that underwent malignant transformation and metastasis. A 6-year-old girl presented with DITs and underwent surgical resection. 12 years later, the tumor recurred and underwent surgical resection again. The pathology report confirmed the lesion to be a glioblastoma multiforme. She received adjuvant treatment. A year after the surgical operation of the lesions, she had intraspinal metastasis and underwent surgical resection again. Multiple spinal cord metastases were subsequently identified in the patient. The patient’s condition exhibited severe deterioration during the follow-up period. This case report focuses on the occurrence of DITs and their potential malignant transformation, as assessed through computed tomography and magnetic resonance imaging.

## 1 Introduction

Desmoplastic infantile ganglioglioma/astrocytoma (DIG/DIA) is a rare intracranial tumor most common in infants under 2 years of age. The tumor is usually located on the surface of the brain. It mainly comprises cystic components with larger volumes and often involves multiple lobes, especially the frontal–parietal lobe. Although this tumor has malignant features on imaging, it was benign and classified as grade I by WHO (1), collectively known as DITs. If surgical resection is complete, the patient has a good prognosis without radiotherapy and chemotherapy. However, the benign nature of this tumor has been questioned with successive reports of atypical, aggressive, and multifocal DITs (2–6). Here, we report a rare case of DITs in a non-infantile who underwent a malignant transformation after surgical resection 12 years later, followed by spread and metastasis.

## 2 Case presentation

**Figure 1:**
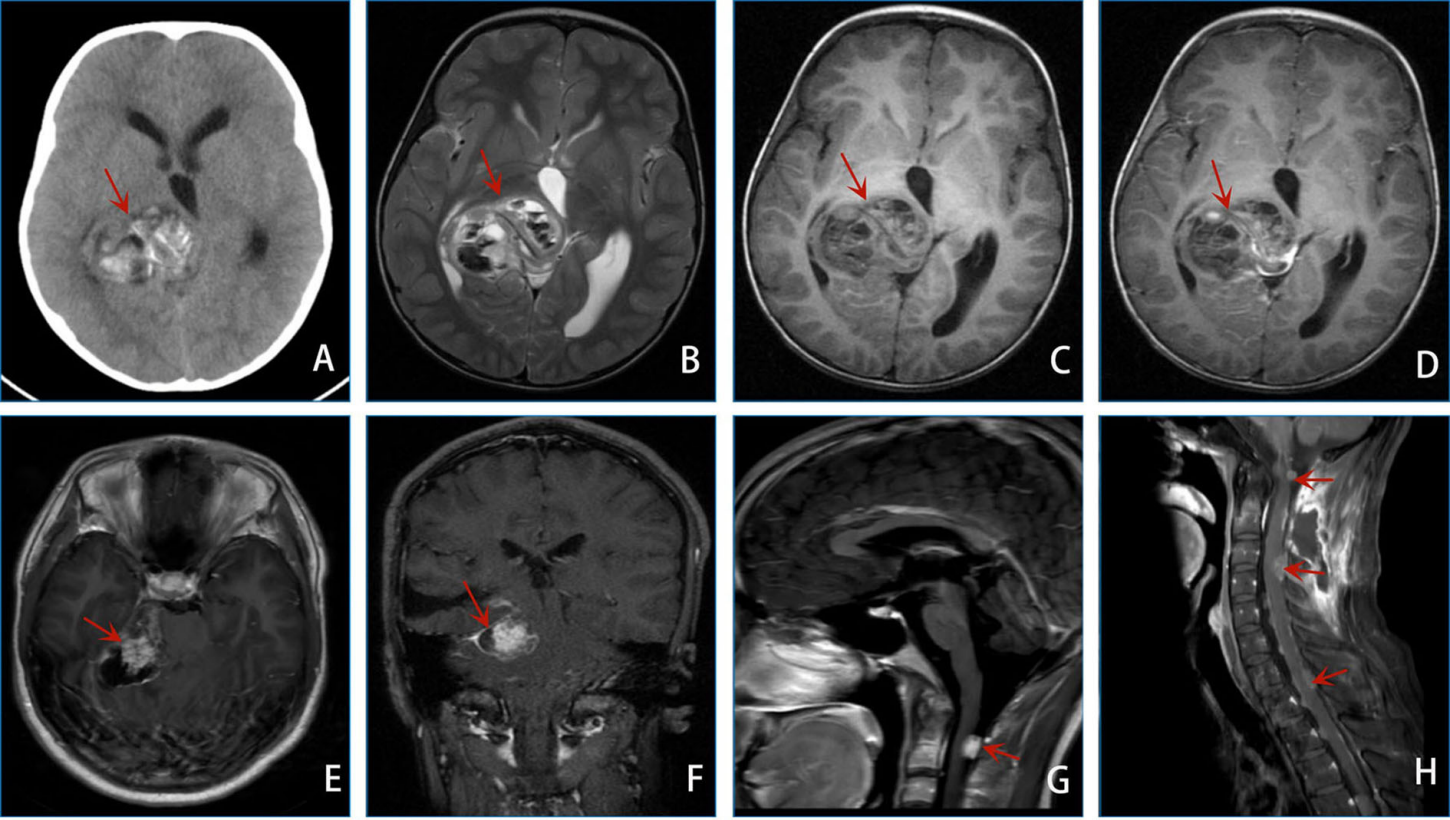
**(A–D)** shows the preoperative image of the patient: CT of the brain **(A)** revealed an irregular jumble density mass with a well-defined boundary of the right thalamus. The right lateral and third ventricles were compressed and deformed, and the center line structure shifted to the left. MRI results of the brain **(B–D)** revealed irregular mixed signals and a remarkably enhancing mass of the right thalamus with multiple cystic lesions and a liquid–liquid plane. **(E–H)** shows the image of the patient 12 years after the operation: MRI results of the brain **(E, F)** revealed some abnormal signals of the medial area of the right temporal lobe, right cerebral foot, cisterna annulus, CPA area, and pontine arm. MRI results of the brain **(G)** revealed a new abnormal enhancing mass of the spinal canal (C1-C2 level). MRI results of the cervical vertebra **(H)** revealed multiple spinal cord spread.

A 6-year-old female patient complained of persistent headaches for 1 year and vomiting for 1 day. The brain computed tomography (CT) scan showed an irregular mass with distinct boundaries in the right thalamus, measuring 54 mm × 43 mm. This mass caused compression and deformation of the right lateral ventricle and the third ventricle, resulting in a shift of the midline structure to the left. Magnetic resonance imaging (MRI) of the brain revealed an irregular mass in the right thalamus, measuring 55 mm × 45 mm × 44 mm, with mixed signals and significant enhancement. Additionally, multiple cystic lesions and a liquid–liquid plane were observed. Immature teratoma or pineal blastoma was diagnosed on the image. Subsequently, she underwent a left temporo-occipital craniotomy with a near-total resection of the tumor. Postoperative pathological showed the mass as a DIG/DIA (**Figure 1**). The patient did not receive adjuvant therapy after surgery.

**Figure 2:**
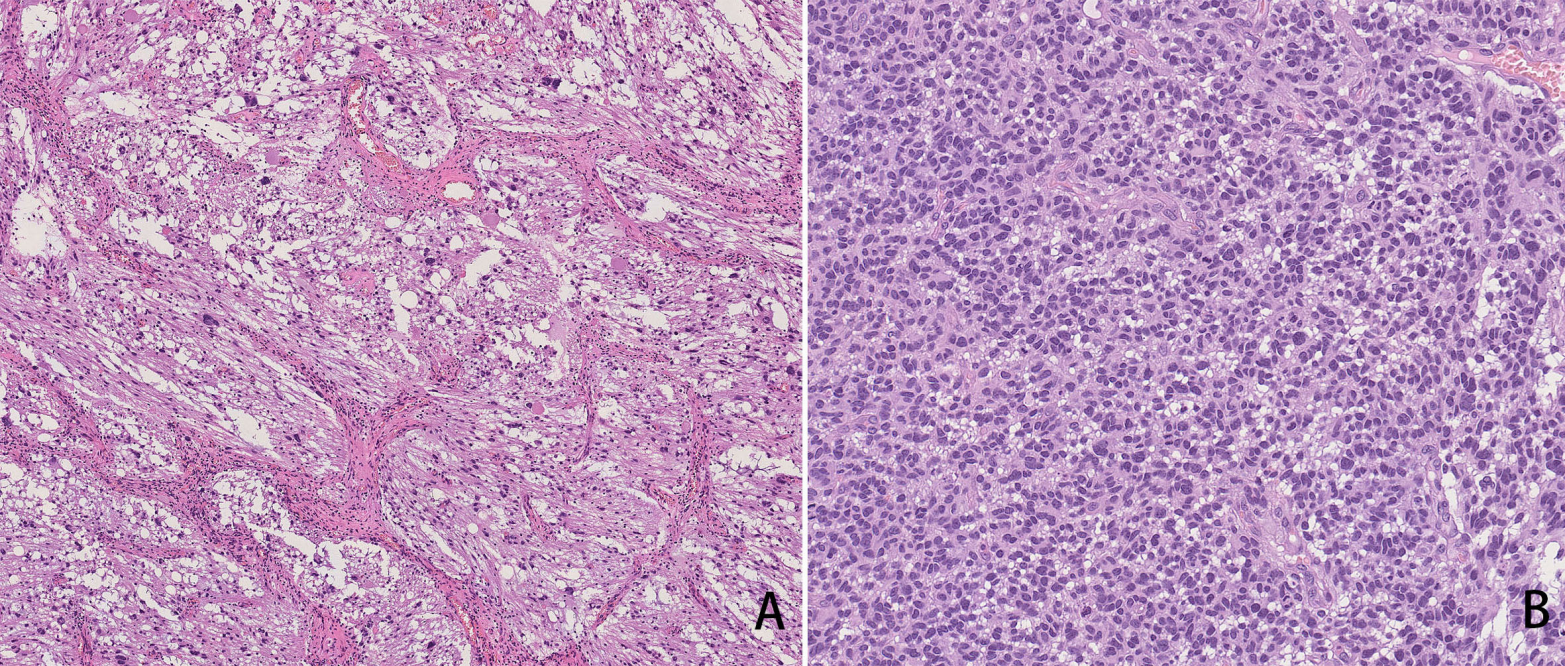
HE staining **(A)**: The tumor was mainly composed of fibroblast-like spindle cells with radial-wheel, bundle, or vortex arrangement and cytoplasmic eosinophilic pleomorphic neoplastic astrocytes with bundle or nest formation distribution. Pathological diagnosis was DIG/DIA. HE staining **(B)**: The tumor cells showed diffuse growth, high density of tumor cells, and prominent atypia. It is easy to see mitotic images, nuclear fragmentation, and local background microcystins. Intravascular glomus hyperplasia was also observed. The pathological diagnosis was glioblastoma (WHO Grade 4).

12 years later, she complained of head and neck pain for 2 weeks, vomiting for 2 days, and dizziness for 2 h. MRI results of the brain revealed some abnormal signals of the medial area of the right temporal lobe, right cerebral foot, cisterna annulus, cerebellopontine angle (CPA), and pontine arm. Tumor recurrence with apoplexy was considered. Subsequently, she underwent right CPA area approach tumor resection. The pathological diagnosis was glioblastoma (WHO Grade 4, **Figure 2**). She received radiotherapy and chemotherapy after surgery.

After the second surgery, 1 year, she developed left lower limb pain and walking instability. Brain MRI revealed a new abnormal enhancing mass of the spinal canal (C1–C2 level), and metastasis was considered. Then, she underwent intraspinal tumor resection. Subsequently, an MRI of the cervical vertebra revealed multiple spinal cord spread. The patient’s condition was terrible with follow-up.

## 3 Discussion

DIA was first reported by Taratuto et al. (7) in 1984. Subsequently, Vandenberg et al. (8) reported the first case of DIG in 1987. DIA and DIG have been classified as grade 1 neuronal and mixed neuron-glial tumors in the WHO due to their similar clinical manifestations, imaging findings, and biological behavior (1). Therefore, they are often described as a whole and called DITs. The tumor is usually located on the surface of the brain and appears as a solid cystic lesion. DITs often occur in infants and rarely in adults (2, 9), which is divided into the infant group (<5 years old) and the non-infant group (>5 years old) (10). These low-grade tumors usually have a good prognosis, but some have malignant features, such as multiple intracranial lesions (3, 4), postoperative recurrence (2, 4, 5), meningeal diffusion, and metastasis (6).

Primary glioblastoma is common, and secondary glioblastoma is rare in children. So far, a few literatures have reported malignant transformation of DITs in infants after tumor resection (11–13), and it is the first case of malignant transformation of DITs in non-infants in our study. We present that a 6-year-old girl with DITs underwent a malignant transformation after surgical resection 12 years later. The tumor’s location and image characteristics differed from those reported in the previous literature. In our study, the tumor was located in the thalamus and cisterna annulus, the brain’s deep part. The tumor was mainly composed of solid components with multiple calcifications and bleeding. It is the first case of secondary glioblastoma of DITs in non-infants. It further validates the notion that DITs have malignant biological characteristics and underscores the importance of careful clinical monitoring during follow-up.

Differential diagnoses based on imaging include primitive neuroectodermal tumor (PNET), supratentorial ependymoma, ganglioglioma, and pleomorphic xanthoastrocytoma (14). PNET is often located in the deep brain, and tumors are mainly presented as solid masses with cystic degeneration, necrosis, and bleeding, with a poor prognosis (15). Supratentorial ependymoma is usually located next to the lateral ventricle triangle, and calcification is common (16). Ganglioglioma often involves the temporal lobe, and the clinical manifestations are complex epilepsy. It is characteristic of calcification in the wall of tumor cysts (17). Pleomorphic xanthoastrocytoma is usually a cystic lesion with enhanced wall nodules. Most patients have a long history of epilepsy (18).

Surgical complete resection is the preferred treatment for the benign biological manifestation of DITs, and the prognosis is usually good without radiotherapy and chemotherapy. Some tumors cannot be resected entirely due to their deep location, and the clinical efficacy of partial resections is poor. Such as our case, the tumor was located in the thalamus and cisterna annulus, the brain’s deep part. It is difficult to complete resection, and the patient underwent a left temporo-occipital craniotomy with a near-total resection of the tumor. 12 years later, the tumor underwent recurrence, malignant transformation, and metastasis. It has been reported that deep tumor location is a factor of increased mortality and an independent predictor of reduced time of tumor recurrence (19). Therefore, postoperative follow-up is necessary for patients with deep tumors and incomplete resection. For patients with residual tumor progression during follow-up, a second surgical resection is required with adjuvant chemotherapy and/or radiation therapy. There is currently no consensus on either chemotherapy or radiation because it is well-known that it has long-term and damaging effects on the developing brain (20). In recent years, many scholars have found BRAF V600E mutations in DTI patients, and the identification of BRAF status provides the possibility of targeted therapy for clinically advanced or unresectable cases after resection, which can be used as an alternative to chemotherapy or radiotherapy (19, 21, 22).

In conclusion, although DITs are benign tumors, their biological behavior is diverse, with recurrence, metastasis, and malignant transformation characteristics. Therefore, postoperative patients must follow up, especially those with deep tumor and partial tumor resection. Once the tumor progresses during follow-up, a second surgery is required and radiation and chemotherapy may be given optionally.

## Data availability statement

The original contributions presented in the study are included in the article/supplementary material. Further inquiries can be directed to the corresponding author.

## Ethics statement

Written informed consent was obtained from the individual(s), and minor(s)’ legal guardian/next of kin, for the publication of any potentially identifiable images or data included in this article.

## Author contributions

YY: Resources, Writing – original draft, Investigation. BT: Data curation, Methodology, Writing – original draft. XC: Formal Analysis, Validation, Writing – review & editing. XL: Supervision, Writing – review & editing. SL: Supervision, Writing – review & editing.
